# Editorial: Novel agents and combinations for treatment of malignant pleural mesothelioma in pre-clinical models

**DOI:** 10.3389/fphar.2022.1015959

**Published:** 2022-09-06

**Authors:** Sze-Kwan Lam, Steven Eugene Mutsaers

**Affiliations:** ^1^ Division of Respiratory Medicine, Department of Medicine, Queen Mary Hospital, The University of Hong Kong, Pokfulam, Hong Kong SAR, China; ^2^ Institute for Respiratory Health, University of Western Australia, Nedlands, WA, Australia

**Keywords:** malignant pleural mesothelioma, novel therapies, novel combinations, animal models, review

Malignant pleural mesothelioma (MPM) is a deadly cancer primarily of the lung pleura, strongly associated with asbestos exposure. According to the ESMO Clinical Practice Guidelines, MPM patients may be suitable for best supportive care only, active (but not multimodality) or multimodality treatments. Surgery, although controversial, is an option in some centres, but the recognised standard of care is systemic chemotherapy (cisplatin-pemetrexed) while other therapies, including immunotherapy (e.g., Nivolumab-Ipilimumab) or combination therapies (e.g., cisplatin-pemetrexed-bevacizumab) are being investigated (Popat et al., 2022). Despite many clinical trials over the last few years (Mutsaers et al., 2020), the quality of life and survival benefit of these treatments is marginal, and therefore novel therapies for MPM are still urgently needed.

The development of novel treatments for MPM requires substantial preclinical data before drugs can enter a clinical trial. Mesothelioma cell lines are extensively used to screen individual or combinations of drugs to determine cytotoxicity, anti-cancer mechanisms and drug resistance, and to identify potentially clinically relevant biomarkers. However, 2D cell culture has limitations as lack of heterogeneity and a tumour microenvironment and acquisition of mutations limit their usefulness (Wilding and Bodmer, 2014). Three-dimensional co-culture and patient-derived organoids are used to screen drug activity in many different cancers, but very few studies have used them to study mesothelioma (Mazzocchi et al., 2018). The most common model used to examine drug effects in MPM is subcutaneous or intraperitoneal inoculation of human or mouse cancer cell lines in immunocompromised or syngeneic mice respectively. More complicated models include patient-derived xenograft models, asbestos-induced murine tumour models and MPM-prone genetically modified mice (Shamseddin et al., 2021). Robinson et al. review their use of different mouse models to investigate therapeutic approaches to treat MPM. They developed some of the first human and mouse mesothelioma cell lines using human tumours in immunocompromised mice and intraperitoneal injection of asbestos respectively. They also developed the MexTAg mouse, an asbestos-induced mesothelioma mouse model in which expression of the Simian Virus 40 large T antigen (SV40 TAg) is controlled by a mesothelin tissue-specific promoter. The advantage with this model is it generates 100% mesotheliomas in response to asbestos. More recently, they also developed a bilateral tumour model which was developed to analyse the difference between immune checkpoint blockade responsive and non-responsive tumours against a homogeneous background.

Key features of cancer, including evasion of apoptosis, unlimited replication, tissue invasion and metastasis, angiogenesis, autocrine growth signals and resistance to anti-growth signals are common targets for cancer therapy (Hanahan and Weinberg, 2000). Pezzicoli et al. discussed current and potentially upcoming standards of care for MPM and reviewed several novel therapeutic agents recently in clinical trial. Lurbinectedin is a transcriptional inhibitor and blocks the function of tumour associated macrophages. Mesothelioma cell lines were highly sensitive to lurbinectedin and in the SAKK 17/16 phase II clinical trial, lurbinectedin demonstrated activity following immune checkpoint inhibitor therapy, indicating a phase III trial is warranted. Pezzicoli et al. also discussed the outcome pf several tyrosine kinase inhibitor trials. Of particular interest, were results from preclinical studies using CDK4/6 inhibitors, that demonstrated cell toxicity and inhibition of tumour growth in mouse models. The MiST2 study (a phase II clinical trial using the CDK4/6 inhibitor abemaciclib, which treated MPM patients deficient in p16ink4A) was recently announced. The median progression free survival and overall survival was 128 and 217 days respectively. The authors plan to further investigate CDK4/6 inhibition in another randomised study (Fennell et al., 2022).

Lapidot et al. reviewed three emerging targets (STAT3, KDM4A, and heparanase) in MPM which have not yet entered clinical trial. STAT3 is important for cell survival, proliferation, self-renewal and chemoresistance. KDM4A demethylates the tumor suppressor SETD2 products H3K36me3 and H3K9me3, which are essential in cell growth and DNA damage repair. Heparanase promotes tumor growth, angiogenesis, metastasis and chemoresistance. All molecules reviewed impact some of the hallmarks of cancers but their role in MPM and the effect of targeting these molecules on MPM tumour growth require considerably more preclinical studies.

Repurposing of existing drugs has also been proposed for MPM. Quinacrine (QC), which is an antimalarial drug and an antibiotic, was shown by Oien et al. to have anti-cancer effects in cisplatin-resistant and pemetrexed-resistant MPM cell lines. Interestingly, NF2-mutated cell lines were more sensitive to QC. Inhibition of the hippo pathway was identified using RNA-seq and closely-associated transcription factors *YAP1*, *CDK4*, and *KRAS* were downregulated. However, the effect of QC *in vivo* has not been investigated.

Precision medicine approaches to treat MPM have been proposed (Quetel et al., 2020; Severson et al., 2020; Meiller et al., 2021) and the use of CRISPR and next generation sequencing (NGS) approaches may help to identify and validate potential targets (Selvakumar et al., 2022). CRISPR can edit genes resulting in gain or loss of function and drug resistance and gene function can be elucidated by CRISPR screening. However, the use of CRISPR and NGS in MPM are far behind studies in other cancer types. In 2021, the presence of ALK gene fusions with alterations in CDKN2A, NF2 and BAP1 (Cordier et al., 2022), alterations in DNA repair genes (BRCA1, BRIP1, CHEK2, SLX4, FLCN and BAP1) (Sculco et al., 2022), fusion of exon 2 of STRN and exon 20 of ALK (Miyagawa et al., 2021) and methylthioadensoine phosphorylase (MTAP) codeletion with CDKN2A (Chapel et al., 2021) were all identified by different NGS platforms and had previously been reported in different pre-clinical studies. However, drugs to modulate many of these targets have not yet been developed for use in clinical trials, highlighting the difficulty of currently applying precision medicine in MPM treatment.

Developing new approaches for treating MPM are urgently needed and it has been suggested that a circular pathway of bench to bedside and back again approach may be key to clinical breakthroughs (Hampton, 2017). Many preclinical studies fail to achieve their full potential in human trials for various reasons such as funding and poor trial design, but a disconnect between basic scientists and clinical researchers may also be a cause. Now more than ever, patient genome data and new technologies such as CRISPR/Cas9 have revolutionised how we can use *in vitro* and *in vivo* models to test gene and variant function. Together, scientists and clinicians can utilise their specific expertise to collaborate and plan better animal studies and clinical trials to maximise effective drug design to treat diseases like MPM which have shown limited therapeutic improvement.
